# Are Thrombolytics Useful in Out-of-Hospital Cardiac Arrest?

**DOI:** 10.7759/cureus.91796

**Published:** 2025-09-07

**Authors:** Gilbert Abou Dagher, Joudie Alwan, Ralphe Bou Chebl, Aseel Sarieddine, Omar Y Fakhreddine, Mohammad Hatoum, Dany Bou Ammar, Ziyad Ghazzal

**Affiliations:** 1 Emergency Medicine, American University of Beirut Medical Center, Beirut, LBN; 2 Pediatric Emergency Medicine, American University of Beirut Medical Center, Beirut, LBN; 3 Cardiology, American University of Beirut Medical Center, Beirut, LBN; 4 Internal Medicine, American University of Beirut Medical Center, Beirut, LBN

**Keywords:** cardiac resuscitation, case report, out-of-hospital cardiac arrest, review article, thrombolytics

## Abstract

Out-of-hospital cardiac arrest (OHCA) is a global health concern with persistently low survival rates. The most common cause of OHCA is coronary heart disease, often leading to abnormal heart rhythms such as refractory ventricular fibrillation (VF). Thrombolytic therapy's role in OHCA, especially in cases secondary to acute myocardial infarction (AMI), remains uncertain.

We present a case of a previously healthy 43-year-old male patient who presented in asystole to the emergency department (ED), following a witnessed cardiac arrest. The patient was intubated and advanced cardiac life support (ACLS) initiated. Systemic thrombolytic (alteplase 100 mg) was administered intravenously 19 minutes into his ED presentation and return of spontaneous circulation (ROSC) was achieved at minute 47 with electrocardiogram (EKG) showing intraventricular conduction delay. The patient was transported to the catheterization laboratory and was found to have a total occlusion of the left obtuse margin artery and a drug eluting stent was deployed within 18 minutes of ROSC. The patient was extubated on day 3 with normal neurological examination and was discharged from the hospital 22 days after admission.

This case builds on the previous literature that systemic thrombolysis may be of added value when used in resuscitation as a salvage therapy in OHCA patients, potentially by lysing coronary thrombi and improving perfusion before PCI. Further studies are needed to shed the light on this important aspect of resuscitation management.

## Introduction

Out-of-hospital cardiac arrest (OHCA) has an incidence of 55-88 per 100,000 worldwide [1]. It remains an important cause of morbidity and mortality as the overall survival to discharge rate following OHCA persists to be low, around 9% in the United States [2]. Survival rates may vary by regions as they are reported to be around 8% in Europe and range between 0.5% and 8.5% in Asia [3-4]. Coronary and structural heart diseases are common underlying causes of OHCA, often leading to abnormal heart rhythms, with ventricular fibrillation (VF) accounting for around 17% of initial rhythms [2]. Early defibrillation is critical for survival; up to 20% of VF patients may progress to refractory VF, which is defined as persistent fibrillation beyond three shocks without achieving return of spontaneous circulation (ROSC) [3]. According to the American Heart Association (AHA) guidelines, the use of medications such as epinephrine, amiodarone, and lidocaine is recommended and has been well-validated by data. The use of thrombolytic therapy, however, remains an area of ambiguity due to insufficient evidence [4]. Thrombolytic therapy has been extensively studied in resuscitation, particularly for pulmonary embolism (PE)-related cardiac arrest, which is endorsed by the AHA [5]. However, its effectiveness for other nontraumatic cardiac arrest causes, such as acute myocardial infarction (AMI), remains uncertain [4]. In 2008, Bottiger et al.'s randomized controlled trial (RCT) challenged earlier positive findings, concluding that thrombolytic therapy during cardiopulmonary resuscitation (CPR) does not improve 30-day mortality outcome for OHCA [6]. Despite this, smaller studies continue to suggest potential benefits [7-10]. We report a case of successful resuscitation, with intact neurological recovery, following refractory VF secondary to coronary heart disease using thrombolytics as salvage therapy.

## Case presentation

A previously healthy 43-year-old male patient presented to the emergency department (ED) by emergency medical services (EMS) following an episode of chest pain and witnessed OHCA with a downtime of 15 minutes, out of which he had received 10 minutes of interrupted low-quality CPR. His initial rhythm at presentation showed asystole. The patient was intubated, and an advanced cardiac life support (ACLS) protocol was immediately initiated. At seven minutes post-arrival, his cardiac rhythm changed to VF. Subsequently, he received seven biphasic shocks of 200 J each. During resuscitation, the patient received three doses of 1 mg epinephrine, 2 g calcium gluconate, 80 mEq of sodium bicarbonate, a total of 450 mg of amiodarone, and 2 g of magnesium sulfate.

Despite the previously mentioned measures, ROSC was still not achieved, and the ED team decided to give thrombolytics for refractory VF in a setting of AMI as the most likely cause of the OHCA. At 19 minutes post-arrival, 100 mg of recombinant tissue plasminogen activator (alteplase) was administered.

This was followed by intravenous administration of six doses of epinephrine 1 mg, lidocaine 100 mg, and amiodarone 300 mg intravenous bolus, along with six biphasic defibrillator shocks of 200 J each, followed by two double sequential defibrillations of 400 J.

**Figure 1:**
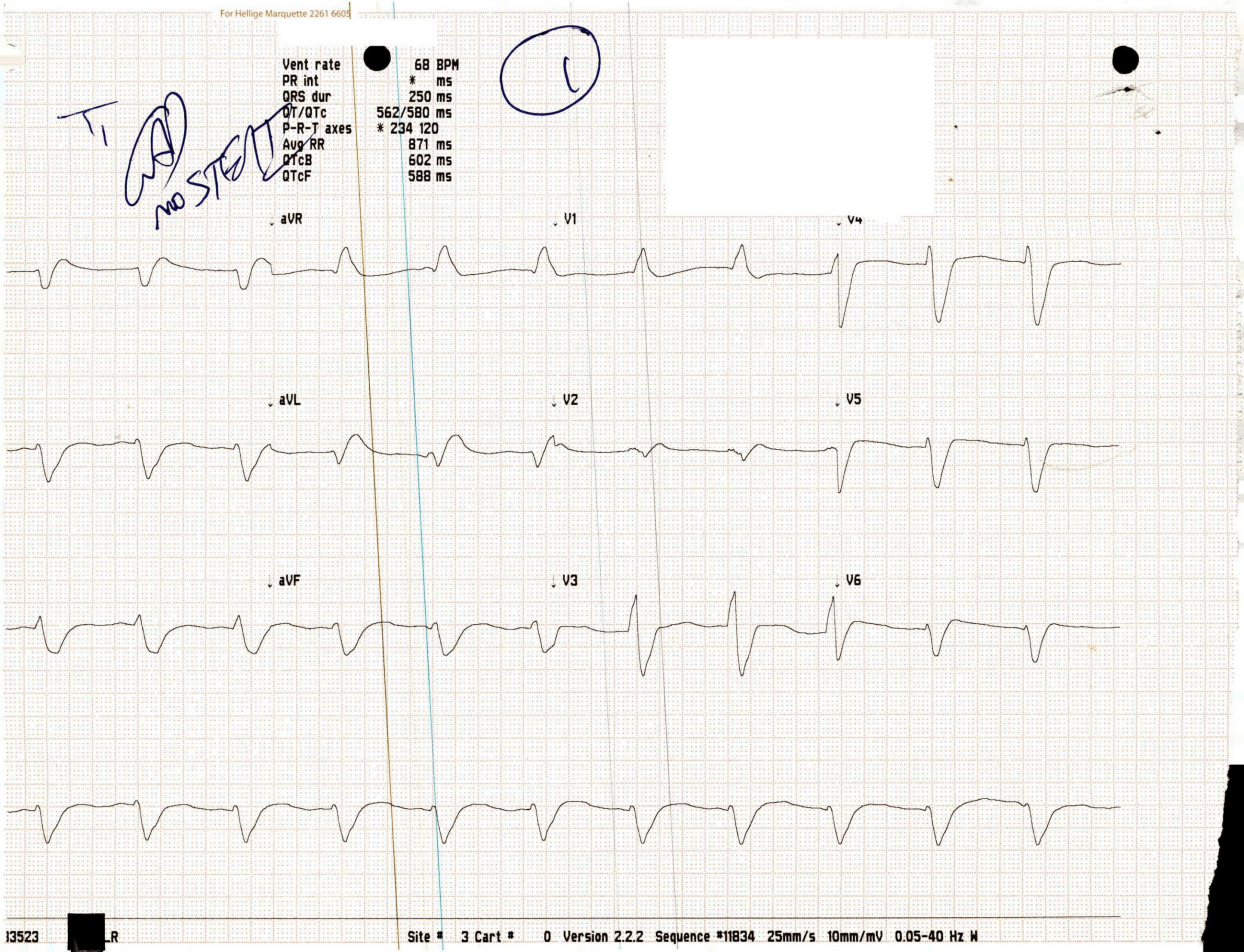
ECG reading post-ROSC achievement showing AIVR ECG: electrocardiogram; ROSC: return of spontaneous circulation; AIVR: accelerated idioventricular rhythm

Patient achieved ROSC at minute 47 from ED arrival and 17 minutes from alteplase administration. Vital signs were the following: pulse (P), 59 bpm; blood pressure (BP), 93/68 mmHg; and mean arterial pressure (MAP), 78 mmHg. The monitor showed sinus bradycardia, and a 12-lead EKG showed intraventricular conduction delay (Figure 1).

Meanwhile, the patient further received sodium bicarbonate 100 mEq intravenous bolus and one dose of epinephrine 1 mg intravenous bolus for sinus bradycardia with a P of 36 bpm and for a BP of 76/40 mmHg, after which his BP stabilized at around 90/50. No spontaneous breathing was noted on the ventilator, and the patient had absent corneal reflexes and fixed and dilated pupils.

**Figure 2:**
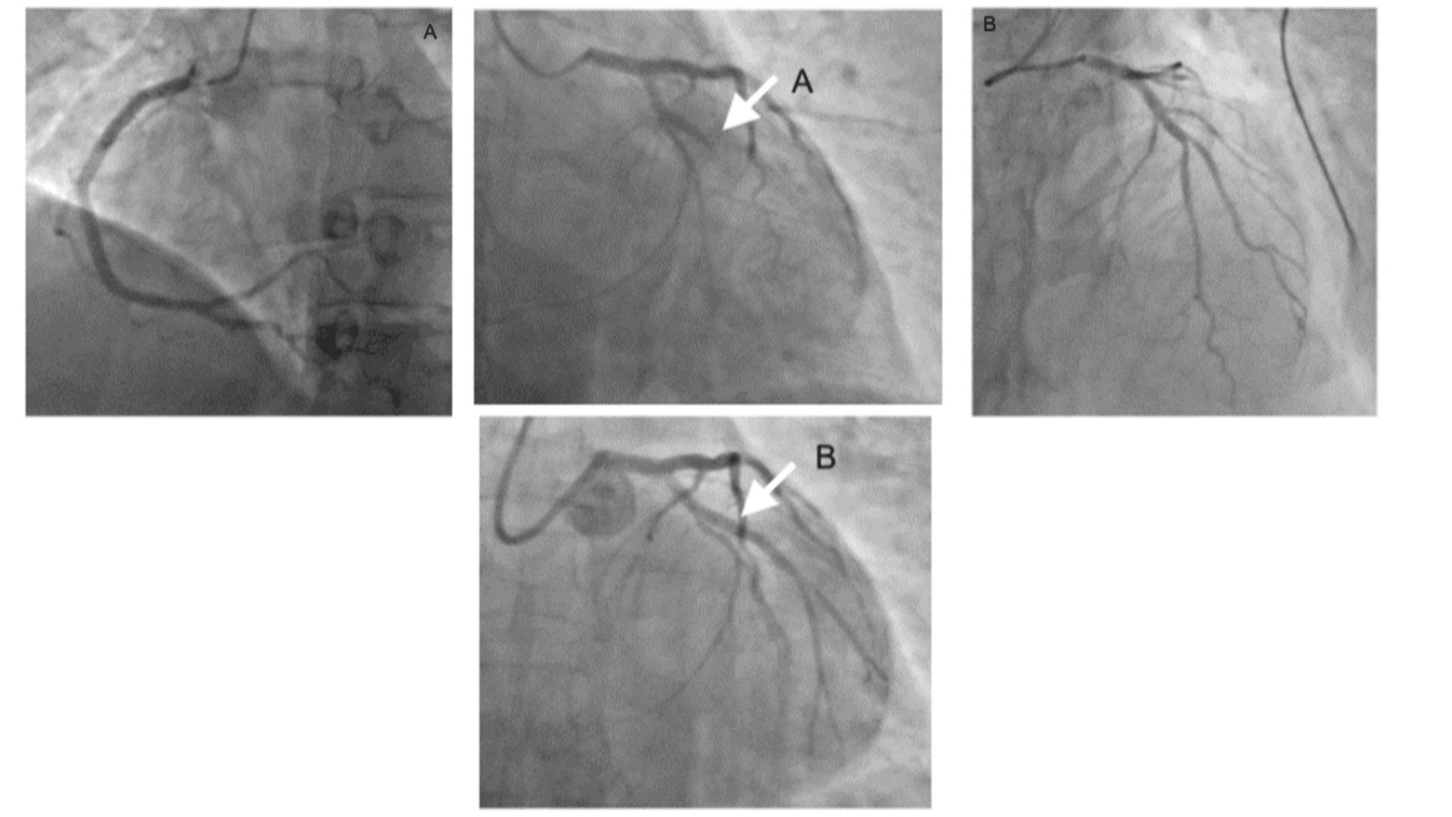
Coronary angiogram pre- and post-stent placement (A) Pre-interventional angiogram showing total occlusion of the obtuse marginal coronary artery. (B) Post-interventional angiogram showing reinstatement of the blood flow to the distal obtuse marginal coronary artery

The cardiology team was consulted, and given the patient’s young age and history suggestive of an AMI, the catheterization lab was activated. In the meantime, the patient was started on an epinephrine intravenous drip (IVD) and an amiodarone IVD, awaiting transport to the catheterization lab. A total occlusion of the left obtuse margin was discovered, leading to the insertion of one drug-eluting stent at minute 65 after arriving at the ED (Figure 2). Subsequently, he was transferred to the cardiac care unit for further postcardiac arrest care.

The hospital stay was notable for a swift recovery period. Neurological status was initially assessed during a sedation awakening trial and showed intact brainstem and cortical neurological function within 36 hours of his presentation. Magnetic resonance angiography of his brain without gadolinium showed no sign of ischemic changes except for a possible small subdural hematoma.

His hospital course was complicated by a mild upper gastrointestinal bleed that was due to friable gastric mucosa on gastroscopy and was treated with proton pump inhibitors (esomeprazole drip). He also developed septic shock from pneumonia that required broad-spectrum antibiotics and vasopressors. His status greatly improved, and he was then extubated with progressive improvement of his mental status back to its baseline without any residual deficit at day 3. He was discharged from the hospital 22 days after admission.

## Discussion

Literature review 

A literature review of the published cases of OHCA secondary to AMI necessitating the use of thrombolytic was performed by searching the following databases: EMBASE, PubMed, Google Scholar, and Medline. Search terms included “OHCA,” “cardiac arrest,” “heart arrest,” “resuscitation,” “cardiopulmonary resuscitation,” “CPR,” “thrombolytic,” “fibrinolytic,” “alteplase,” and “tissue plasminogen activator.”

The literature yielded 13 published papers of non-traumatic OHCA secondary to AMI necessitating the use of thrombolytic therapy. The study selection was accomplished through two steps. First, the literature was reviewed, and all papers that describe patients of interest were included. Afterward, full articles were obtained for all accepted studies. Excluded articles included those that had OHCA secondary to PE, those that received thrombolytic therapy after ROSC was achieved, analytical articles that did not differentiate between PE or AMI as a presumed cause of OHCA, or articles that were published in any language other than English (due to translation limitations).

The 13 obtained articles were comprised of five analytical papers, two case series papers, and six case reports. Methodological quality was not formally assessed, as most included studies were case reports/series with inherent heterogeneity.

The main findings of the included papers are summarized in the tables. Table 1 presents demographics, details on thrombolytic usage, bleeding complications, and neurological outcomes in the case reports and case series. Table 2 offers insights into the durations of different resuscitative phases within the case reports and case series. Likewise, in these papers, Table 3 examines the ECG rhythm recorded both before and after thrombolytic administration. Table 4 describes the same variables in analytical papers found in the literature.

**Table 1:** Description of population in case reports and case series ROSC: return of spontaneous circulation; F: female; M: male; TPA: tissue plasminogen activator; CPC: cerebral performance category; * missing data (1) Neurological outcome described based on the cerebral performance category score ‡ No CPC score as patient died on day 4

Authors	Number of patients	Age	Gender	Agent	Dose	ROSC	Bleeding	Neurological outcome (1)
Tiffany et al. [11]	3	41	F	TPA	*	Yes	No	CPC 1
		57	F					CPC 1
		49	M					CPC 1
Duchateau et al. [12]	1	53	F	TPA	80 mg	Yes	No	CPC 1
Fraipont et al. [13]	1	72	M	Reteplase	20 IU	Yes	Yes: mediastinal hematoma	‡
Aliyev et al. [14]	4	52	F	Streptokinase	500,000 IU bolus then 1,000,000 IU infusion	Yes	No	CPC 1
		58	M			Yes		CPC 1-2
		63	M			Yes		CPC 1
		42	M			No		*
Foley et al. [15]	1	58	M	Tenecteplase	*	Yes	Yes: tongue hematoma	CPC 1
Kim et al. [9]	1	43	M	Tenecteplase	40 mg	Yes	No	CPC 1
Archan et al. [10]	1	48	M	Tenecteplase	80 mg	Yes	No	CPC 1
Hamera et al. [7]	1	47	M	TPA	50 mg	Yes	No	CPC 1

**Table 2:** Different time points in resuscitation in case reports and case series CPR: cardiopulmonary resuscitation; ROSC: return of spontaneous circulation; *missing data; ^‡^not applicable, but total duration of resuscitative efforts lasted 150 min (1) The time between the onset of CPR and ROSC

Authors	Time from CPR initiation to thrombolytic administration (minutes)	Time from thrombolytic administration to ROSC (minutes)	Low flow time (1) (minutes)
Tiffany et al. [11]	8	4	12
	21	9	30
	17	5	22
Duchateau et al. [12]	60	15	75
Fraipont et al. [13]	*	*	*
Aliyev et al. [14]	30	5	35
	60	15	75
	60	20	95
	45	‡	‡
Foley et al. [15]	*	*	*
Kim et al. [9]	25	45	70
Archan et al. [10]	28	5	68
Hamera et al. [7]	57	11	68

**Table 3:** ECG rhythms depending on thrombolytic administration time in case reports and case series VF: ventricular fibrillation; VT: ventricular tachycardia; PEA: pulseless electrical activity; MI: myocardial infarct; *not reported

Authors	Before thrombolytic administration	After thrombolytic administration
Tiffany et al. [11]	VF/VT	Sinus
	VF/VT	Sinus
	Pulseless VT	Sinus
Duchateau et al. [12]	VF	Sinus
Fraipont et al. [13]	VF	Inferior MI
Aliyev et al. [14]	Asystole	VF then sinus with anterior MI
	VF then asystole	Sinus with inferior right MI
	Asystole	Anteroseptal MI
	Electromechanical dissociation	*
Foley et al. [15]	VF	Sinus
Kim et al. [9]	Asystole then VF	PEA then sinus
Archan et al. [10]	Asystole	VF
Hamera et al. [7]	VF then PEA	Anterolateral MI

**Table 4:** Analytical papers (1), (2) ROSC: return of spontaneous circulation; TPA: tissue plasminogen activator; CPC: cerebral performance category; *missing data (1) Low flow time: time between the onset of CPR and ROSC. (2) Age and low flow time: presented either as mean ± SD or mean (min-max)

Authors	Number of patients receiving thrombolytics	Age	Male gender (%)	Agent	Dose	Low flow time (min)	ROSC (%)	Bleeding	Neurological outcome	Notes
Bottiger et al. [16]	40	64 ± 10	27/40 (68%)	TPA	50 mg	40 ± 23	27/40- 68%	2/27-7.4%	*	Bleeding not related to CPR (P=0.379)
Schreiber et al. [17]	42	53 (46–66)	32/42 (76%)	TPA	100 mg	12 (9 – 22)	100%	*	CPC 1-2: 69%	-
Kurkciyan et al. [18]	132	55 (47-64)	103 /132 (78%)	TPA	100 mg	12 (4 – 24)	100%	10 - 13%	CPC 1-2: 52%	Survival at 6 months: 63%
Richling et al. [19]	101	53 (46-62)	82/101 (81.2%)	Reteplase or	100 mg or	17 (10 – 28)	100%	*	CPC 1: 56%	The bleeding complication rate was comparable with that of patients who were not thrombolysed
				Tenecteplase	Weight-based					
Koeth et al. [20]	107	64 (53 – 72)	*	*	*	*	101/107 – 94%	*	*	-

In Tables 1, 4, neurological outcome is measured by the cerebral performance category to unify the results from the literature whenever available; however, some studies lacked standardized outcome measures, and missing data were noted in the tables. The score ranges from 1 to 5, with 1 indicating good cerebral performance with full recovery or mild disability and 5 indicating brain death.

Discussion

Refractory VF is defined as fibrillation that persists beyond 3 shocks without achieving ROSC [3]. Refractory VF is more resistant to therapy than non-refractory VF due to more severe underlying myocardial pathology, greater electrical instability, and higher ischemic burden, often from acute coronary occlusion [21]. Patients with initial shockable rhythms that turned into refractory VF had a linear decline in probability of survival rates as well as favorable neurological outcomes [22]. Dealing with a case of OHCA refractory VF in a relatively young patient can be challenging as to when one should withhold all resuscitative efforts. According to AHA ACLS protocols, the main resuscitative interventions include but are not limited to high-quality CPR, antiarrhythmic medications, vasopressor medications (epinephrine), and early defibrillation. 

The use of thrombolytic therapy in resuscitation has been a subject of study for many years, notably more studied for cardiopulmonary arrest secondary to pulmonary embolism. As per the AHA, thrombolytic therapy is endorsed for cardiac arrest resulting from pulmonary embolism. Nevertheless, its application remains uncertain for other causes of non-traumatic cardiac arrest, especially in the context of acute myocardial infarction (AMI) [4]. Thrombolytic use in AMI-related OHCA has been particularly understudied since the 2008 Thrombolysis in Cardiac Arrest (TROICA) trial showed no survival benefit, compounded by bleeding concerns and primary percutaneous coronary intervention (PCI) becoming the standard reperfusion strategy [6].

The literature yielded a total of 13 patients from a combination of case reports and case series [7,9-15]. The mean age of the patients studied is 52.5 years. There was a lack of consistent reporting on the dosage and specific thrombolytic agent utilized across these cases. ROSC was achieved in 92% of the cases, with most patients reporting a favorable neurological outcome classified as Cerebral Performance Category (CPC) score of 1, except for two cases that did not survive. Similarly, our patient was discharged with CPC 1. The average time from CPR initiation to thrombolytic administration was 37.36 minutes, while the mean duration from thrombolytic treatment to achieving ROSC was only 13.4 minutes. In contrast, the total duration of resuscitative efforts averaged 63.63 minutes. These findings suggest the importance of considering prolonged CPR in these cases. Our patient’s total resuscitation time lasted 47 minutes, and ROSC was achieved 17 minutes following tissue plasminogen activator (TPA) administration. While this prolonged resuscitation was feasible in our tertiary care center, it is important to note that such extended efforts may not be possible in many EDs due to staffing, space, and resource limitations

Multiple studies, including Li et al.’s meta-analysis, have demonstrated an increased rate of ROSC in patients receiving thrombolytics, particularly in those with prolonged resuscitation [11,16,23-27]. As opposed to that, in 2008, Bottiger et al. published the TROICA study, the first double-blinded placebo-controlled RCT, challenging these findings by concluding that thrombolytic therapy during CPR does not improve outcomes for OHCA [6]. This contradicted the previously published positive results [11,16,23-27]. The authors of the RCT also reported an increased rate of intracranial hemorrhage, with a rate of 2.7%. In our patient’s case, although a minor subdural hematoma was present on imaging, it was not clinically significant. It is worth mentioning that the trial differentiated between four types of hemorrhage**,** and while the trial noted a significant increase in intracranial hemorrhage, symptomatic intracranial hemorrhage did not reach statisticalsignificance. The negative result in the TROICA trial might be attributed to the absence of adjunctive antithrombotic or antiplatelet agents. This could explain why the RCT was suspended after futility analyses and why the authors concluded that Tenecteplase (without adjunctive antithrombotic medications) during CPR does not improve outcome; however, this remains a hypothesis. Further limitations included scarce in-hospital care information, which could have affected the primary outcome of 30-day survival. It is important to note that the RCT did not differentiate between PE vs. non-PE etiology of cardiac arrest. Survival data may be susceptible to performance bias as the authors allowed the open-label use of thrombolytics for suspected OHCA secondary to PE. This could have excluded a subgroup of patients from randomization who eventually could have benefited from thrombolytic medication [6]. Following this RCT, the AHA issued its 2010 guideline, which recommended against routine use of fibrinolytics during cardiac arrest but allowed their use when PE is suspected as the cause of cardiac arrest [4]. Subsequent meta-analyses, which included both AMI and PE as the causes of cardiac arrest, also failed to demonstrate any statistically significant improvement in outcomes for patients treated with thrombolytics. It's worth mentioning that these meta-analyses were heavily weighted by the TROICA trial data, likely due to its large sample size and study design [28,29]. It's important to emphasize that existing literature does not provide evidence of thrombolytic use being linked to adverse outcomes; rather, the available papers consistently report no difference in survival rates. Conversely, one case report and one retrospective study indicated once more that thrombolytic therapy was associated with increased survival to hospital admission, notably when employed as a salvage therapy [7,8]. This can imply that thrombolytics may offer additional benefits when used as adjuvant treatment during resuscitation rather than serving as the definitive solution for AMI. Notably, they may enhance overall neurological outcomes, especially when utilized as a salvage therapy in prolonged resuscitation cases. Further supporting this perspective, in the TROICA trial, the median time from CPR initiation to achieving ROSC was 22 minutes in the treatment group and 25 minutes in the placebo group. However, it's crucial to acknowledge that this ideal scenario might not be universally applicable, as observed in other countries or settings. As previously noted, this duration extended to an average of 50.76 minutes in cases retrieved from the literature, aligning closely with the 47 minutes observed in our patient. This may suggest a potential benefit of thrombolytics in prolonged resuscitation scenarios, shedding light on why they might not have demonstrated efficacy in the RCT.

During cardiac arrest and resuscitation, there is an imbalance in coagulant and anticoagulant factors, leading to a procoagulant state and microthrombosis formation. This process contributes to increased mortality and morbidity by decreasing perfusion to crucial organs. One of the reasons why TPA is thought to be beneficial in OHCA is the medication’s action of restoring microcirculatory reperfusion and enhancing prognosis by decreasing tissue injury.

Previously, the administration of TPA was discouraged due to concerns about potential bleeding complications caused by CPR. However, numerous studies have since demonstrated the safety of using TPA, effectively dispelling these concerns [11,16,30].

Only two case reports stated having bleeding complications (Table 1) [13,15]. It is important to highlight that these two papers lacked critical information regarding thrombolytic dose, timing of administration, and total resuscitative time. Likewise, analytical papers in the literature lack consistency in describing the selection of thrombolytic agents and their dosages [6,16-20]. The absence of standardized definitions for bleeding complications further contributes to the inconsistent reporting of bleeding complications across these studies. Our patient, like previous reported case reports, did not suffer from CPR-related hemorrhage.

Schreiber et al. first investigated neurological outcomes in patients receiving thrombolytic therapy during cardiac arrest as a result of AMI [17]. He reported that TPA was associated with “favorable neurological outcomes in patients after ventricular fibrillation cardiac arrest due to AMI” even after controlling for confounders [17]. This was reinforced by subsequent studies that confirmed either normal neurological function or minimal disability after the administration of thrombolytic medication [26,31,32]. Our patient was discharged with full neurological recovery following resuscitation without any residual disability, aligning with the findings reported in the literature. We could not find in the literature any reports of severe disability (CPC 3-4) from our search.

Despite this comprehensive review, several limitations should be considered when investigating the application of thrombolytics in OHCA. Notably, many studies were excluded because they did not differentiate between the cardiac and pulmonary embolism etiologies of OHCA. Furthermore, the role of physicians in these studies may introduce detection bias, as their initial decision to administer thrombolytics could be influenced by assumptions regarding the cause of OHCA or better prognostic factors related to patient selection. The lack of standardized medication and dosage usage across studies could also impact the observed results. These limitations underscore the need for further research and the establishment of standardization of protocols to provide clearer guidelines for the use of thrombolytics in OHCA scenarios.

## Conclusions

This case adds to the growing body of anecdotal evidence suggesting potential utility of thrombolytic therapy as a salvage treatment in non-traumatic OHCA, particularly in patients presenting in refractory VF. Although alteplase did not result in immediate reperfusion of the infarct-related artery, it may have contributed to ROSC and favorable neurological recovery by improving microcirculatory perfusion or resolving distal coronary microthrombi. The absence of major bleeding complications in this case also supports the potential safety of thrombolytic use in this context. However, as a single case, it cannot establish causality, and the observed benefit must be interpreted with caution. This underscores the urgent need for controlled studies that address key knowledge gaps, including the optimal timing and dosing of thrombolytics in OHCA, the identification of patient subgroups most likely to benefit, and the potential role of adjunctive antiplatelet or antithrombotic therapies to enhance efficacy and reduce risk.
